# Annual periodicity in planktonic bacterial and archaeal community composition of eutrophic Lake Taihu

**DOI:** 10.1038/srep15488

**Published:** 2015-10-27

**Authors:** Junfeng Li, Junyi Zhang, Liyang Liu, Yucai Fan, Lianshuo Li, Yunfeng Yang, Zuhong Lu, Xuegong Zhang

**Affiliations:** 1 MOE Key Lab of Bioinformatics; Bioinformatics Division/Center for Synthetic and Systems Biology, TNLIST and Department of Automation, Tsinghua University, Beijing, China; 2 State Key Lab for Bioelectronics, School of Biological Science and Medical Engineering, Southeast University, Nanjing, China; 3 Wuxi Environmental Monitoring Centre, Wuxi, China; 4 State Key Joint Laboratory of Environment Simulation and Pollution Control, School of Environment, Tsinghua University, Beijing, China; 5 Department of Biomedical Engineering, Peking University, Beijing, China

## Abstract

Bacterioplankton plays a key role in nutrient cycling and is closely related to water eutrophication and algal bloom. We used high-throughput 16S rRNA gene sequencing to profile archaeal and bacterial community compositions in the surface water of Lake Taihu. It is one of the largest lakes in China and has suffered from recurring cyanobacterial bloom. A total of 81 water samples were collected from 9 different sites in 9 different months of 2012. We found that temporal variation of the microbial community was significantly greater than spatial variation (adonis, n = 9999, *P* < 1e−4). The composition of bacterial community in December was similar to that in January, and so was the archaeal community, suggesting potential annual periodicity. Unsupervised K-means clustering was used to identify the synchrony of abundance variations between different taxa. We found that the cluster consisting mostly of ACK-M1, C111 (members of acIV), *Pelagibacteraceae* (alfV-A) and *Synechococcaceae* showed relatively higher abundance in autumn. On the contrary, the cluster of *Comamonadaceae* and *Methylophilaceae* (members of lineage betI and betIV) had higher abundance in spring. The co-occurrence relationships between taxa were greatly altered during the cyanobacterial bloom according to our further network module analysis.

In recent years, eutrophication and consequential algal blooms in aquatic systems have become a major environmental issue that alters ecosystems and threatens human lives^1^,^2^. Recurring algal blooms can be found in freshwater systems all over the world, such as Lake Victoria in Africa^3^, Lake Erie in North America^4^, and Lake Taihu in China^5^. A number of studies have been carried out to unveil the mechanism of bloom formation, understand the consequences on ecosystems and find possible solutions. It is widely agreed that over-enrichment of nutrients from anthropogenic sources especially nitrogen and phosphorus promote the development of algal blooms^6^,^7^,^8^,^9^,^10^. Besides, other environment factors such as temperature, pH, and day length may also influence the bloom formation^9^,^11^,^12^. Toxic materials such as microcystin may be generated during or after bloom and consequently alter the community structure of the aquatic ecosystem^13^,^14^. Bacterioplankton is a focus of study because it is closely linked to phytoplankton bloom^15^,^16^,^17^ and plays a key role in nutrient cycling^18^,^19^. However, time dynamics of the bacterioplankton community in shallow eutrophic lakes remains unclear.

Lake Taihu, with an area of 2,338 square kilometers and an average depth of 1.9 meters^20^, is the third largest freshwater lake in China. It is a typical shallow eutrophic lake and has been suffering from severe cyanobacterial bloom in recent years. Previous studies on Lake Taihu mainly focused on analyses of environmental factors. It has been reported that cyanobacterial bloom usually initiates in spring when the temperature gradually rises^21^,^22^. Total nitrogen (TN) and total phosphorus (TP) are control factors of bloom formation and different ratios of TN and TP have different promotion effects^23^,^24^,^25^. Those aimed at microbial community were mainly based on coarse-resolution technique like PCR-DGGE^26^,^27^,^28^,^29^,^30^,^31^,^32^,^33^, T-RFLP^34^,^35^,^36^. Dominating bacteria in both sediment and water are *Alpha-, Beta-, Gamma-* and *Deltaproteobacteria, Actinobacteria* and *Bacteriodetes*. The archaeal community varies in different layers of sediment and water, and mainly consists of *Eruyarchaeota* and *Crenarchaeota*^32^. Ammonia-oxidizing archaea (AOA) has been found to be negatively correlated with the accumulation of organic substances^37^. Several microcystin-degrading bacteria have been identified and isolated^38^,^39^.

To obtain a high-resolution profile of the microbial community, we used high-throughput next-generation sequencing (NGS) to profile the bacterial and archaeal community compositions on a monthly basis for a year at multiple locations of Lake Taihu, using our strategy of next-generation sequencing targeting 16S rRNA gene^40^. We are specifically interested in the following scientific questions: (i) What the temporal patterns of bacterial and archaeal community compositions are; (ii) How the location variation of community composition is when compared to temporal variation; (iii) How the co-occurrence relationship between microbes changes with the development of cyanobacterial bloom.

## Materials and Methods

### Sample Collection and 16S rRNA Gene Sequencing

**Figure 1:**
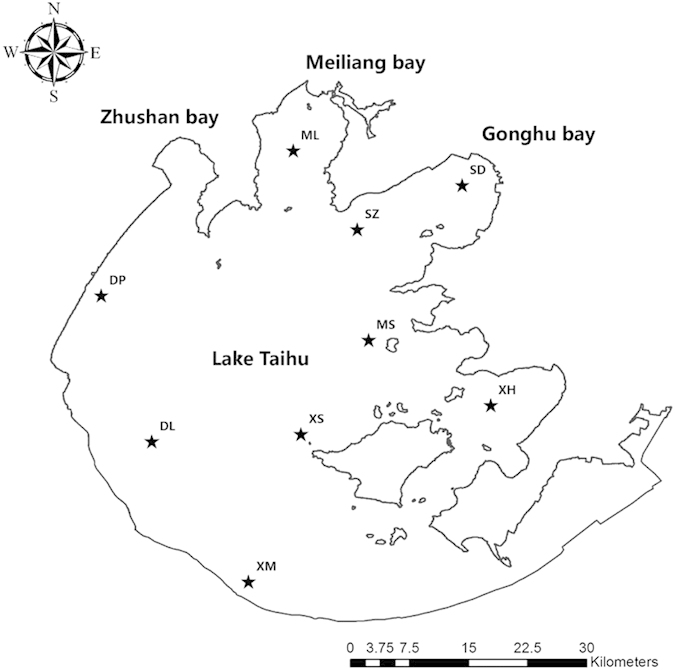
Geographic location of 9 different sample sites in Lake Taihu. Solid stars denote locations of the 9 national monitor stations where the samples were collected in this study.

To examine temporal and spatial variations of the microbial community, freshwater samples were collected at 9 sites in 9 months during 2012 (Fig. 1 & Table 1). Specifically, 2 *l* of original water sample at the depth of 0.5 m was pre-filtered through a steel mesh with ~0.015 mm diameter pore size to remove large particles. We also took efforts to remove bloom species (mainly *Microcystis* here) in water samples, as otherwise they would consume most of sequencing capacity and affect the profiling of the microbial community. This was done by re-filtering the water samples through a filter with 0.22 μm diameter pore size to collect microorganisms. These filters were stored at −20 °C until further molecular analysis.

DNA samples were extracted from the filters using E.Z.N.A. ® Water DNA Kit (OMEGA, USA). The quality and the quantity of DNA were examined by agarose gel electrophoresis and spectrophotometrically quantified by Nano Drop ND 2000 (Thermo Scientific, DE, USA). Then the DNA was used as the template for amplifying the V6 region of 16S rRNA genes. PCR primers validated in the literature^41^ were chosen for bacteria and archaea respectively (Table S1). Barcodes and linkers were designed and embedded into PCR primers following our published protocol^40^. To extract archaeal V6 region, PCR amplification was initiated by a denaturation cycle of 96 °C for 5 min, followed by 25 cycles at 96 °C for 45 sec, 48 °C for 45 sec, 72 °C for 1 min, and a final extension step at 72 °C for 10 min. For the amplification of bacterial V6 regions, the following PCR condition was used: 96 °C for 5 min; then 16 cycles of 96 °C for 45 sec, 48 °C for 45 sec, 72 °C for 1 min, and, finally, 72 °C for 10 min. The second round of PCR amplification was implemented for bacteria and archaea to introduce Illumina sequencing adapters. Similar PCR condition was used: 98 °C for 30 sec; then 9 cycles of 98 °C for 10 sec, 65 °C for 30 sec, 72 °C for 30 sec, and, finally, 72 °C for 10 min. PCR products of the archaea and bacteria DNA were mixed at the ratio of 1:5 for subsequent sequencing according to the pre-experiment that we designed to estimate the ratio. The Illumina HiSeq 2000 platform was used to sequence paired-end (PE) reads with length 100 bp in both forward and reverse directions.

In order to measure the influence of embedded barcodes on PCR, we used primers with different barcodes to amplify one sample selected from the collected samples and compared the results. To verify the consistency and reproducibility of experiment results, 56 out of 81 samples were also sequenced by Illumina GAIIx platform using 2*120bp paired-end strategy as replicates.

### Data Preprocessing

Forward-end reads and reverse-end reads of the raw sequencing data were matched and joined using PANDAseq^42^, which was helpful to reduce the error rate of sequencing reads and elongate the length of Illumina reads^40^. Reads containing ambiguous ‘N’ or with length <120 nt or >140 nt were discarded. Subsequently, FASTX Toolkit^43^ was applied to trim the barcodes and linkers from the remained joined reads. Sequences were assigned to corresponding samples according to the trimmed barcodes, and those with unmatched barcodes were discarded. Archaeal and bacterial reads were distinguished based on their different PCR primers using customized Perl scripts and then analyzed separately. Quality control was carried out with the FASTX Toolkit, and filtering parameters (Archaea: 90% bases quality score >30; Bacteria: 100% bases quality score >30) were chosen based on the results of FastQC^44^. This is a stricter criterion compared to similar studies, ensuring the high quality of the results.

### OTU Clustering and Taxonomy Assignment

The QIIME platform v1.8.0^45^ was applied in the subsequent data processing after quality control. Reads were then clustered into species-level OTUs (operational taxonomic units) at 97% similarity, using the subsampled open-reference-based OTU-picking workflow in QIIME based on UCLUST^46^. The Greengenes database (version 13_5) was used as the reference^47^. Chimera reads and the corresponding OTUs were removed by ChimeraSlayer^48^ and QIIME scripts. We chose 0.001% as the threshold for filtering low-abundance OTUs, i.e., only OTUs with read counts >0.001% of the total reads of all samples were kept. UCLUST consensus taxonomy assigner was applied in the taxonomic information assignment for the remained OTUs. The most specific taxonomic labels associated with at least 51% (QIIME default) of database hits of OTU reads were assigned to the OTU. Representative reads for OTUs were picked using default settings in QIIME and then aligned to the Greengenes database by PyNAST^49^. In the Greengenes database, chloroplast is listed as a class belonging to *Cyanobacteria* and contains orders such as *Chlorophyta, Cryptophyta, Haptophyceae* and *Stramenopiles* that are actually eukaryotes. Therefore, we did not include them in the downstream analysis since our study focused on prokaryotes. Phylogenetic trees were constructed based on the aligned reads using FastTree^50^.

### Microbial Diversity and Statistical Analysis

Microbial diversity was measured by a series of OTU-based analyses of alpha- and beta-diversity implemented in the QIIME pipeline. For the alpha diversity, rarefaction curves were drawn based on two richness metrics, “observed species” and “PD_whole tree”^51^, and two evenness metrics, Shannon entropy and Simpson metric^52^. We chose a sequencing depth that most samples were at the plateau of rarefaction curves and explored microbial richness using R scripts. For beta diversity, phylogenetic-based Unifrac metric^53^ and OTU membership-based dissimilarity Jaccard metric^54^ were employed to measure the pairwise community similarity between samples that were re-sampled to equal sequencing depth. Emperor^55^ was used to visualize the distance matrix of all the 81 samples based on PCoA (Principle Coordinate Analysis). The hierarchical clustering method UPGMA was applied to group samples according to their distance matrix; the resulting tree file was visualized by FigTree v1.4.0 as well as the phylogenetic tree^56^. Significance of the differential taxa between UPGMA groups was tested by Kruskal-Wallis rank-sum test. The threshold was set as Bonferroni corrected *p*-value < 0.05. Adonis^57^ implemented in QIIME was used to test whether differences among groups of samples were significant. Mantel test^58^ was used to test whether the β-diversity distance matrices of samples from HiSeq 2000 was significantly correlated with those from GAIIx. K-means clustering of genera based on relative abundance was performed using R scripts. We firstly resampled the original OTU table for normalizing the sequencing depth, which ensured every sample had the same number of total reads. Since OTUs were annotated to species level, we summed abundance of OTUs affiliated with the same genus and used it as the abundance of that genus. For a certain genus *G* of all identified genera, relative abundance in a certain sampling month *T* was calculated as formula (1).


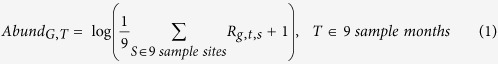


Here, *R*_*g,t,s*_ is the resampled read count of genus *G* in the sample collected at site *S* in month *T*. Logarithmic read counts was used as the measure of relative abundances. 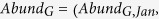

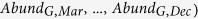
 was used to denote abundance of the genus *G* across 9 sampling months. For comparison of temporal variation, we transformed 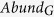 of each genus into Z-score using formula (2),


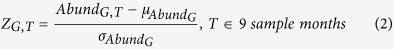


where 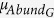 and 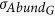 denote the mean and standard deviation of *Abund*_*G*_, respectively. Transformed Z-score of all the genera were calculated as described above. To determine the appropriate number of clusters, we used the ratio of within-cluster sum of squares error (SS) to between-cluster SS as a measurement of clustering performance. To reduce biases caused by randomness of the K-means algorithm, we used 100 random sets of initial centers and reported the result with the best clustering performance.

### Co-occurrence Network Analysis

Co-occurrence and mutual exclusion information of OTUs has been widely used to predict species interactions in environments^59^. The 81 samples were clustered into groups based on the UPGMA result, and we constructed an OTU co-occurrence network for each group. Network nodes represent OTUs, and edges represent co-occurrence or mutual exclusion relationship between OTUs. Pearson Correlation Coefficient (PCC) was adopted to measure the strength of the relationship as used in the literature^60^,^61^,^62^. OTUs that existed in less than half of the samples within a certain group were removed to reduce false-positives caused by excessive mutual exclusions. To this end, we firstly calculated pairwise similarity and generated similarity matrix, and used the scale-free property as a criterion for determining the threshold since scale-free is a major characteristics for biological networks^60^,^61^,^63^,^64^, which indicated that a few OTUs held most edges and many other OTUs were rarely linked. Cytoscape^65^ was employed to visualize the resulting networks. We used MCODE plugin^66^ to find network modules in one network and trace them in the others.

## Results

### Sequencing data analyses and microbial community characterization

We explored the microbial community of Lake Taihu via targeted 16S rRNA gene sequencing. By different experimental settings, we generated three datasets: (I) All the 81 samples sequenced by Illumina HiSeq2000; (II) one of the 81 samples was amplified with 14 different barcodes, generating 14 samples sequenced by Illumina HiSeq2000; and (III) 56 of the 81 samples sequenced by Illumina GAIIx. Dataset-I was the main data source of this study, while Dataset-II and Dataset-III were used to validate our PCR primers and reproducibility of the experiments, respectively. A single run of HiSeq2000 generated 1,046,311,536 paired raw reads. After PE reads joining and barcode parsing, 955,690,310 reads remained and were filtered with strict quality control criteria (see Materials and Methods for details). We finally obtained 705,037 ± 345,118 archaeal reads and 3,241,126 ± 1,114,800 bacterial reads per sample, which was about 1:5 as the experiment design. We had done pre-experiment and found that rarefaction curve of archaea and bacteria diversity can both level off even at the archaea/bacteria concentration ratio of 1:10 (data not shown). Therefore, the present 1:5 ratio was chosen in order to pay more attention on the minority archaea while the sequencing depth has been guaranteed. More details about the preprocessing are shown in Table S2. The ChimeraSlayer output showed that there were no chimera reads using the Greengenes database (gg_13_5) as reference.

**Figure 2:**
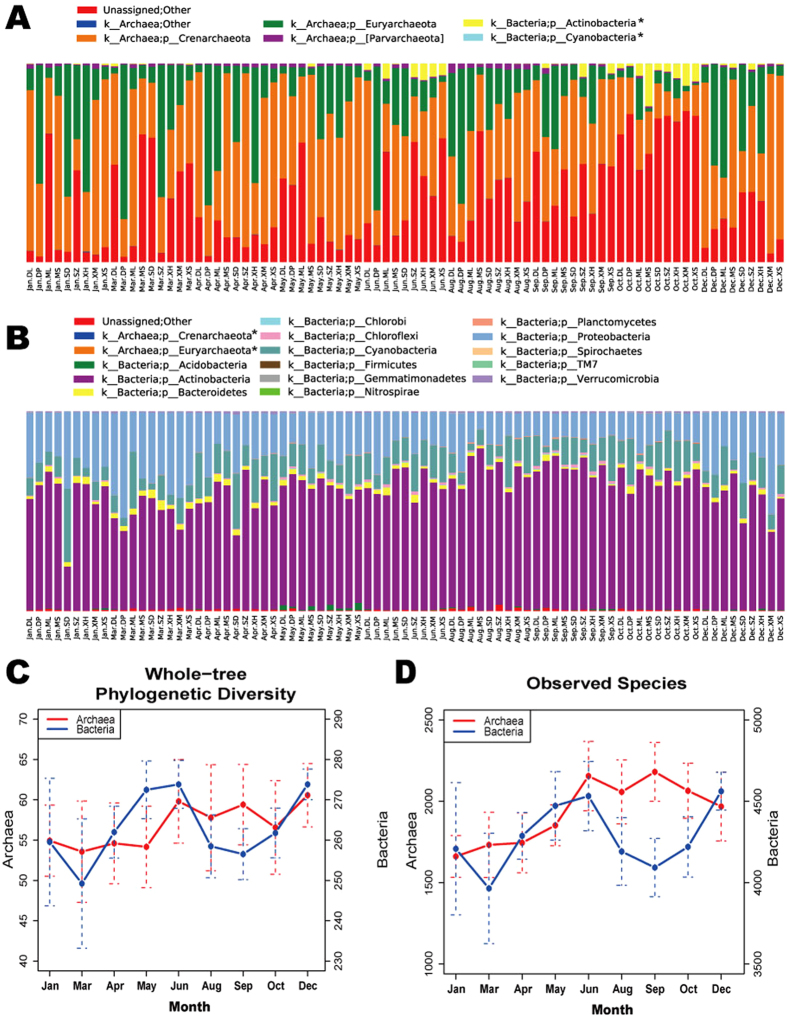
Phylum-level microbial community profile and species richness of archaea and bacteria. (**A,B**) represent the relative abundances of bacterial and archaeal phyla in the 81 samples, respectively. Sample IDs are composed of the sampling month and site name. Bar colors represent different phyla, and bar lengths represent the relative abundances. The bacteria and archaea phyla marked with “*” include taxonomies that have highly similar 16S rRNA sequences and are not distinguishable by the PCR primer. (**C,D**) show temporal variations of archaeal and bacterial richness within samples, measured with two metrics, whole-tree phylogenetic diversity and observed species, respectively. Sequencing data were firstly resampled to the same sequencing depth for all samples. The left y-axis and right y-axis are for archaeal and bacterial curves, respectively.

Based on these high-quality non-chimeric reads, we finally obtained 3,046 archaeal OTUs and 5,295 bacterial OTUs. Taxonomic information of archaea and bacteria OTUs were assigned based on the Greengenes database. We found that there were some bacteria in the archaeal profile and vice versa, which was probably due to limited specificity of PCR primers or the indistinguishability between some archaea and bacteria. We discarded those OTUs from this study. As shown in Fig. 2, dominant (relative abundance >1%) archaeal phyla were *Crenarchaeota* (43.8 ± 22.6%), *Euryarchaeota* (22.0 ± 21.1%) and *Parvarchaeota* (22.0 ± 21.1%). Dominant bacterial phyla were *Actinobacteria* (60.7 ± 10.2%), *Proteobacteria* (21.9 ± 9.6%), *Cyanobacteria* (12.5 ± 6.5%) and *Bacteroidetes* (2.1 ± 0.9%). A large portion of archaeal reads remained unassigned (31.2 ± 22.5%), while unassigned bacteria reads accounted for only 0.7 ± 0.5%.

Further analysis at lower taxonomic level showed that *Nitrosopumilus* was the most abundant genus in *Crenarchaeota* (20.7 ± 18.6%). *Methanosaeta* and an unclassified genus of candidate family *Methanomassiliicoccaceae* represented the most abundant *Euryarchaeota* genera with average abundance 5.0 ± 7.0% and 5.5 ± 5.1%. For bacteria, *Betaproteobacteria* (15.4 ± 10.8%)*, Alphaproteobacteria* (4.9 ± 3.2%) and *Gammaproteobacteria* (1.5 ± 1.3%) represented the most abundant groups of *Proteobacteria.* An unclassified genus of ACK-M1 family (clade acI-A) was the dominants of the community across all samples, accounted for 48.1 ± 8.0% of total abundance. Abundance of different genera of *Proteobacteria* were relatively evenly distributed. The most abundant one was an unclassified genus of *Pelagibacteraceae* family (clade alfV-A, tribe LD12) with 4.5 ± 3.2% of total abundance, while members of *Comamonadaceae* (lineage betI, 6.2 ± 4.6%) and *Methylophilaceae* (lineage betIV, 3.5 ± 4.4%) represented the most abundant groups of *Betaproteobacteria. Gammaproteobacteria* were widely regarded as temporary members from anthropogenic or zoonotic sources. It’s reasonable for them to show a low abundance in freshwater, mainly *Xanthomonadaceae, Pseudomonadaceae* and *Moraxellaceae* (clade gamV, gamIV and gamIII). More details about the abundance profiles are listed in Tables S3 & S4. Here, we need to point out that these community profiles could be biased due to our pre-filtering operations. However, since we carefully chose the pore size of filters in order to keep most prokaryotes, such bias will not have significant influence on our conclusions.

### Primer validation and experiment reproducibility

**Figure 3:**
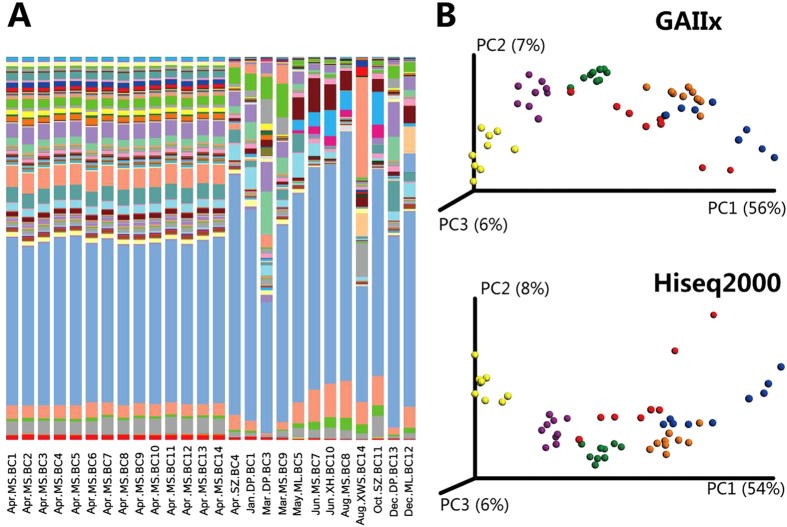
Difference of the bacterial community structure and β-diversity between samples with different PCR barcodes and sequencing platforms. (**A**) Genus-level taxonomy profile of samples using different PCR barcodes. Sample IDs are composed of sampling month, site and barcode index. Legends of the bar colors are omitted in this figure as the purpose is to check the consistence between samples using different barcodes. Details of genera are listed in supplementary Table S6. (**B**) PCoA results of 56 samples sequenced by the GAIIx platform and the Hiseq2000 platform based on relative abundance of OTUs using unweighted Unifrac metric.

Use of different barcodes in PCR primers may have an impact on PCR amplification that may cause bias in species abundance measurements. To check this issue, the “Apr.MS” sample was chosen to be sequenced using different barcodes (BC1-BC14). Using the same data processing protocol for Dataset-I, we generated Dataset-II and summarized the preprocessing information in Table S5. Figure 3A shows that genus-level taxonomic profiles were highly consistent among the Apr.MS samples with different barcodes (details in Table S6), but significant variations can be found among other samples. We concluded that the influence of different barcodes on the taxonomic profiles can be ignored.

Different sequencing platforms may also cause biases. To check this issue, 56 samples were chosen to generate extra sequencing replicates by the Illumina GAIIx platform, leading to Dataset-III. We summarized the preprocessing information in Table S7. Based on the unweighted Unifrac metric, distributions of sample points sequenced by the GAIIx platform and the HiSeq2000 platform were largely consistent (Fig. 3B). Furthermore, Mantel test showed that the distance matrixes generating two PCoA figures were significantly correlated (*r* = *0.95, p-value* = 1e−4, two-sided, permutation n = 9999). We concluded that our results were independent on sequencing platforms.

### Temporal and spatial variations of microbial diversity

We explored microbial diversity within samples (α diversity) by rarefaction curves (Fig. S1). Two diversity metrics defined in QIIME, “observed species” and “PD_whole_tree”, were used as measurements. We can see that rarefaction curves nearly level off, suggesting that we had captured most of abundant microbes. Figure 2C,D show the numbers of observed archaea and bacteria species and PD_whole_tree metric across the sampling months. It demonstrated that there was one peak in the archaeal curve of microbial diversity while there were two peaks in the bacterial curve of microbial diversity.

**Figure 4:**
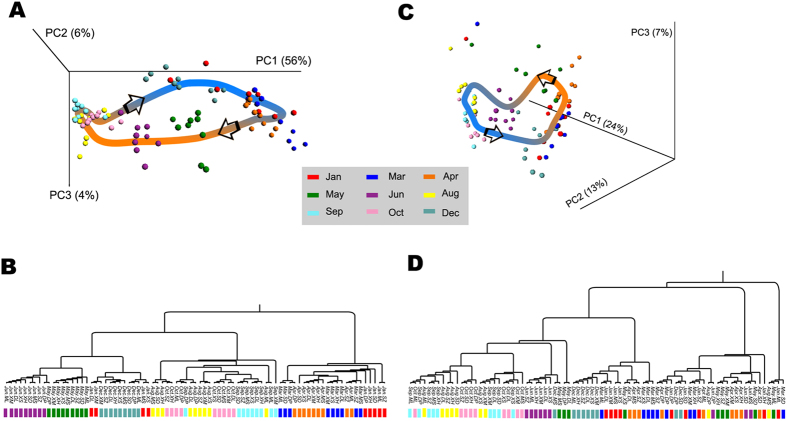
PCoA and UPGMA on bacterial community showing significant clustering correlated with temporal variations. (**A**) PCoA result based on OTU relative abundances of samples using unweighted Unifrac metric. Temporal variation is the main factor contributing to the community variation. Additionally, samples from January to December are distributed in a circled pattern, which implies an annual cycle of the temporal variation of microbial community. (**B**) UPGMA result based on the unweighted Unifrac metric used in PCoA. The hierarchical clustering structure helps to determine the similarity of the microbial communities between different months. Consequently, we group samples into four stages that are correlated with four seasons in a year.

To examine whether variations across sampling sites were greater than variations across sampling time, we pooled all 81 samples and used PCoA and UPGMA to conduct unsupervised clustering analysis. We found that samples collected from the same month were located close to each other on the PCoA plot (Fig. 4A), suggesting that spatial variations were smaller. This was verified by adonis showing that variation across time was more significant (*p-value* = 1e−4, R = 0.27, permutation n = 9999) than that across different sampling sites (*p-value* = 0.6668, R = 0.09, permutation n = 9999). Interestingly, we found that the microbial community varied from month to month gradually, which suggested a trend of community succession. Especially, we observed that the community in December was more similar to that of January, which contributed to the ring-shaped PCoA plot. Considering that environmental conditions like temperature, which is usually the main factor contributing to community variation, are quite similar in the same month, it is reasonable to infer that community in December will be more similar to that in the next January. This suggests a potential annual periodicity of the community variation in Lake Taihu. Also, previous studies based on multi-year data have reported that seasonal patterns in bacterioplankton community structure are reoccurring in freshwater systems^67^,^68^,^69^, which supports our conclusion.

Based on UPGMA result (Fig. 4B), these samples could be clustered into four stages: Dec. ~ Jan., Mar. ~ Apr., May ~ Jun., and Aug. ~ Oct. (adonis *p-value* = 1e−4, R = 0.57, n = 9999) in line with the four seasons, which also supports the annual periodicity conclusion. The temporal variation was less significant in archaea (adonis *p-value* = 1e−4, R = 0.22, n = 9999) compared to that of bacteria (Fig. 4C,D) considering the r^2^ which showed the percentage of variation explained by the supplied grouping factor. However, the site variation of archaeal community was significant (adonis p-value = 1e−4, R = 0.19, n = 9999). Finally, a total of 73 taxa were identified to be significantly different between the Mar. ~ Apr. stage and the May ~ Jun. stage (Kruskal-Wallis rank sum test, Bonferroni corrected *p*-value < 0.05), while the numbers for May ~ Jun. versus Aug. ~ Oct., Aug. ~ Oct. versus Dec. ~ Jan., and Dec. ~ Jan. versus Mar. ~ Apr. were 42, 24 and 26, respectively (Fig. S2). Further studies about biological functions of these taxa may help us understand the characteristics of the four stages.

### Bacterial community shift and season-specific genera

**Figure 5:**
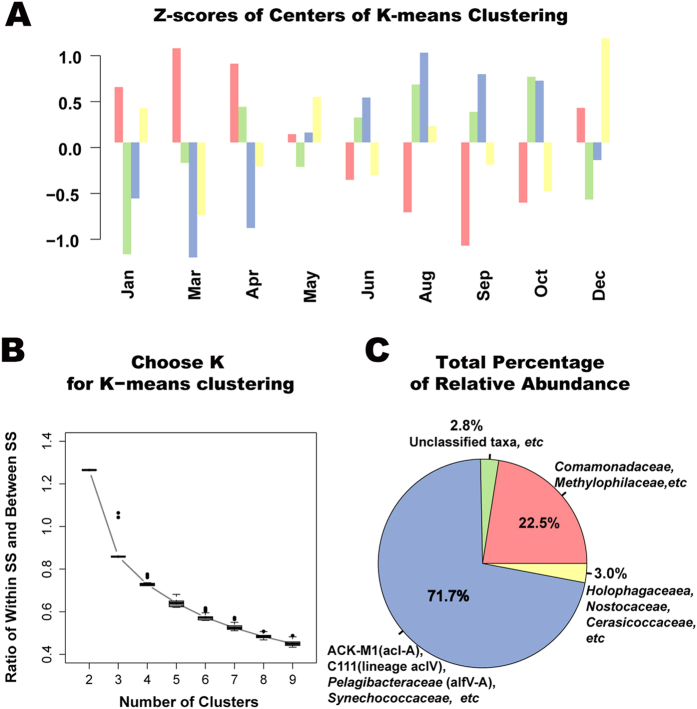
K-means clustering results identifying synchrony of different genera. Relative abundance of genera was firstly transformed into Z-scores as described in Materials and Methods. Then, synchronized genera defined as genera that have similar temporal variations were identified by K-means clustering. (**A**) Relative abundances of “center” genera of resulted clusters at different sampling months. (**B**) The ratio of within-cluster SS and between-cluster SS as function of the number of clusters. This curve helps to determine the appropriate parameter *K* in K-means clustering. (**C**) Total abundance of each cluster and corresponding representation phylum. Phylum assignments of most abundant genera that take up >50% of cluster abundances were used to represent each cluster.

We explored variations of taxa abundance. The variation of abundance can be considered as a kind of response of microbes to the environment change, which possibly reflects the important biological function of taxa. Therefore, it is reasonable to assume that taxa holding the same temporal variation pattern (we call them synchronized taxa) may have similar niche preference or have similar function in the community. Using the K-means algorithm in R to cluster the genera based on their relative abundances across 9 months measured by Z-scores, we grouped taxa with similar changes across the months. We focused on the bacterial community since seasonal variation of archaeal community was less significant and bacteria was much more abundant and had better annotation. We set the number of clusters as 4 according to Fig. 5B with the method described in Materials and Methods.

Figure 5A shows temporal variations of Z-scores of four cluster centers represented in red, blue, green and yellow, respectively. The relative abundance of the red cluster was substantially higher in December to April than in June to October, while the blue cluster displayed the opposite pattern. Thus, we named them as the “spring-specific” cluster and the “autumn-specific” cluster, respectively. There were 84 genera in the “spring-specific” cluster and 71 genera in the “autumn-specific” cluster. These clusters were fairly stable across different sample sites in Lake Taihu as shown in Fig. S3. The “spring-specific” and “autumn-specific” clusters accounted for 22.5% and 71.7% of total abundance, respectively, and the green cluster and the yellow cluster accounted only for 2.8% and 3.0% with 25 and 46 genera, respectively (Fig. 5C). We used a set of most abundant genera within each cluster (of which the sum abundance accounted for more than 50% of the cluster abundance) as the representatives of that cluster (Fig. 5C). We found that representatives were different for 4 clusters. Families ACK-M1 (acI-A), C111 (members of acIV), *Pelagibacteraceae* (alfV-A) and *Synechococcaceae* represented most abundant members of the “autumn-specific” cluster, while *Comamonadaceae* and *Methylophilaceae* (members of lineage betI and betIV) were most abundant in the “spring-specific” cluster. Most members of the green cluster and the yellow cluster were either poorly annotated or annotated to minor phyla such as *Acidobacteria, Verrucomicrobia*. The estimation of minor phyla abundance was less accurate as it is vulnerable to artificial factors in experiments or dataset noise, which might explain the confusing temporal pattern of these two clusters. More details about cluster composition were showed in supplementary Table S8.

### Co-occurrence Network Module Variations

To explore microbial co-occurrence relationship over time, we built four co-occurrence networks based on correlations of relative abundance of OTUs across different sample sites in the four seasons with the same threshold of 0.90 (Table S9). The *r*^2^ of regression was 0.80 ± 0.07 and the exponent of “power-law” was 1.50 ± 0.05, suggesting that the networks were scale-free.

**Figure 6:**
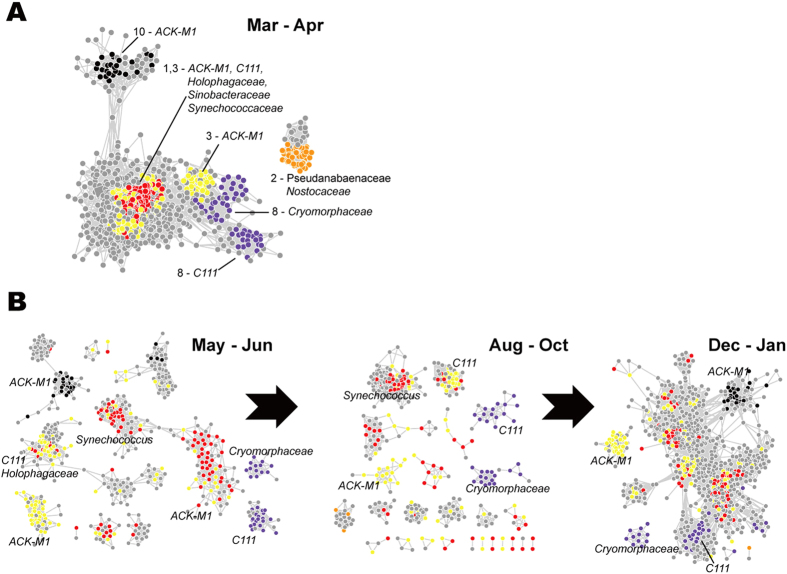
Temporal variations of co-occurrence network modules and relationship between them. Network nodes are OTUs; edges indicate co-occurrence or mutual exclusion relationships between nodes. Modules found by MCODE in the network of the Mar.-Apr. stage and their first-neighbor nodes are demonstrated in (**A**); same OTUs belong to the modules are traced in other three stages as in (**B**). Modules were annotated using taxonomic names at the most specific level.

Topological properties of networks substantially changed over time (Fig. S4). Network density (the average number of edges per node) was higher in Dec.-Jan. (8.37) and Mar.-Apr. (8.6) than that in the other two stages (5.67 in May-Jun. and 5.56 in Aug.-Oct.), suggesting fewer co-occurrence relationships during the period of time of most algal blooms. The difference of bacterial communities between spring and autumn was most significant. We identified modules in the network of the Mar.-Apr. stage using MCODE and traced the top 10 modules ranked by the average degrees (Fig. S4). Figure 6 illustrates the modules and the first neighbors of the nodes of modules in the networks of the four stages. Interestingly, modules were often comprised of OTUs affiliated with only one or a few families (Fig. 6A,B). For Module-1 and Module-3, most OTUs were members of ACK-M1 (acI-A), C111 (members of acIV), *Holophagaceae, Sinobacteraceae* and *Synechococcaceae* (mostly *Synechococcus* genus). For Module-2, OTUs were mainly affiliated with *Pseudanabaenaceae* (mainly the *Leptolyngbya* genus) and *Nostocaceae*. We also found that different modules might share same families, like ACK-M1 in Module-1,-3 and -10 or C111 in Module-3 and -8. It could be possibly attributed to the different genus composition within the same family. When comparing the four networks, we observed that connections between modules were changing significantly over time. For example, members of Module-1 and Module-3 were highly connected in the Mar.-Apr. network, but those connections were gradually lost in the following May-Jun. network and Aug.-Oct. network. Furthermore, module-2 presented in the Mar.-Apr. network even totally disappeared in later time of the year. It’s hard to give the ecological explanations about these patterns based on only taxonomic information. We will further discuss about it and propose our hypothesis in the discussion section.

## Discussion

Based on ultra-deep sequencing data targeting the V6 region of microbial 16S rRNA genes, we profiled temporal and spatial variations of archaeal and bacterial communities in Lake Taihu, which was prone to severe cyanobacterial bloom. Relative abundances of taxa were studied at different taxonomic levels. We observed that a large portion of archaeal OTUs were left “unassigned”. Recent discovery of ammonia-oxidizing archaea greatly broadened the knowledge of prokaryotes functioning in ammonia oxidation, which is closely related to nitrification and thus the eutrophication. Previous studies has revealed ammonia-oxidizing archaea in sediment samples of Lake Taihu^32^,^37^. In shallow lakes like Taihu, surface sediment has intensive exchange with upper water. The microbial community within sediment and water are highly associated. Therefore, more attention needs to be paid to archaea in water besides the sediment, especially the “unassigned” part. Rarefaction curves demonstrated that taxa profile of samples from different sites and different months had revealed most of abundant species within freshwater in Lake Taihu. With the results of re-sequencing part of total 81 samples, we confirmed the reliability and reproducibility of our analysis results when different barcodes and sequencing platforms were used. *Cyanobacteria* was not detected as the most abundant phylum due to the filtering operations during sample collection (see Materials and Methods). Although it may alter the original community structure, such filtering was necessary to make sure that rare taxa could be also covered during the sequencing. Otherwise, most of the sequenced reads would belong to the members of *Cyanobacteria*.

The temporal variations of α-diversity of archaeal communities and bacterial communities were different. The observed two peaks of bacterial richness around May and December could correspond to the recruitment phase and dormancy phase of bloom development^10^. Many genera of *Cyanobacteria* especially *Microcystis* are sensitive to temperature variations and exhibit optimal growth rates at relatively high temperature^1^. When temperature rises in early spring, *Cyanobacteria* recruits and increases the concentration of dissolved oxygen through the photosynthesis. Microorganisms in water increase rapidly at this time as environment conditions are suitable for their growth, which contributed to the first peak in diversity curves. But, as *Cyanobacteria* such as *Microcystis* dominates the freshwater community very quickly and even forms water bloom, other bacteria can be strongly inhibited and even gradually die away because of toxic microcystin. This leads to the decrease of diversity. For the later peak of the diversity, it could be attributed to the recovery and growth of other bacteria because of the dormancy of the dominating *Cyanobacteria*. When temperature is low, the growth of *Microcystis* is strongly inhibited. But for the other bacteria, some prefer relatively low temperature. The dormancy of the dominating *Microcystis* in Lake Taihu gives room for the growth of such kind of bacteria. Therefore, it leads to the increase of microbial diversity. Archaeal diversity showed only one peak that lasted from May to December, which might be attributed to the ability of surviving in low oxygen conditions and the higher temperature optima of archaea^70^,^71^,^72^,^73^. Even during cyanobacterial bloom when the dissolved oxygen were easily exhausted by the excessive bloom species, archaea will not sharply die away so that diversity of archaeal community would have less variation. Basically, archaeal communities in the water body of Lake Taihu were more stable against the influence of cyanobacterial blooms than that of bacteria.

We observed annual periodicity of temporal variations in both bacterial and archaeal communities. Previous studies has reported the existence of seasonal pattern and the annual cycle in microbial community structure of other aquatic systems^67^,^69^,^74^,^75^. There are some studies on temporal variations of biochemical factors or specific species in Lake Taihu^76^,^77^,^78^,^79^,^80^, but not of the microbial communities. In our study, we revealed that both the archaeal and bacterial communities held significant temporal variation and potential annual periodicity. The annual periodicity was less significant in the archaeal community than that of the bacterial community, which may attribute to the significant difference of archaeal community across different geographical locations as aforementioned. On the other hand, previous studies have shown that archaea in sediments and water are quite different while bacteria are not^32^. Therefore, the extensive vertical motion between different layers of water and sediment can greatly alter archaeal community composition and thus disturb the annual pattern. For the annual periodicity, we may speculate that there is a special “original status” of the community, which is the beginning and the ending of the cyanobacterial bloom at the same time. During the development of bloom, the community becomes unbalanced from the “original status” and forms the water bloom. After the end of the bloom, the community tends to restore to the vulnerable “original status” until the next water bloom. Unfortunately, it’s difficult to identify the driven taxa contributing to the annual periodicity based on present experiments. In our future work, we should try to identify such “original status” first by special experiment design and use functional information to study the ecological mechanism.

We identified the synchrony of bacteria using K-means clustering and explored changes of co-occurrence between taxa by reconstructing network modules. It suggested that abundances of most taxa follow a specific variation pattern rather than irregular changes in this eutrophic lake with cyanobacterial bloom. For the “spring-specific” cluster, the most abundant OTU affiliated with *Comamonadaceae* was an unclassified genus. Considering the large diversity of *Comamonadaceae* family, we couldn’t get more specific information for explaining the high abundance in spring. However, *Methylophilaceae* only includes four formally described genera. In the “spring-specific” cluster, the most abundant OTU affiliated with *Methylophilaceae* was an unclassified genus, while the second is the genus *Methylotenera*. According to a very recent study^81^, *Methylotenera* is most similar to tribe LD28 among four genera and LD28 tends to have higher abundance in Mar.-Apr. and Nov.-Dec. This is consistent with the variation of “spring-specific” cluster. Also, it pointed out that the maximum of LD28 in that period was probably mainly determined by the C1 substrates released by phytoplankton. Considering the close relatedness between *Methylotenera* and LD28, we speculated that the variation pattern of the “spring-specific” cluster may also be determined by the same factors. For the “autumn-specific” cluster, recent metagenomic and single-cell genomic studies have reported the living preference of acI-A for N-rich compounds and their potential ability to degrade cyanophycin^82^,^83^. As the most abundant family within the “autumn-specific” cluster, ACK-M1 is affiliated with acI-A and should reasonably be expected to show the characteristics described above. Therefore, it could take good advantage of the large amount of cyanophycin provided by excessive *Cyanobacteria* to acquire energy and carbon during autumn. Consequently, the large proliferation of ACK-M1 (acI-A) demonstrated high abundance in that period. Besides, some previous studies also observed that C111 (members of acIV) and *Pelagibacteraceae* (alfV-A) showed high abundance in autumn. But there were still little knowledge about the ecological functions of these taxa explaining their abundance variation patterns. Further studies about genomic functions are needed.

Network module analysis provided the overview of the change of coexistence and mutual exclusion association between taxa. Since network modules were OTUs that had significant co-occurrence relationships that showed correlated abundance in different environment situations, it is expected that these OTUs would potentially have similar functions or be ecologically interacting with each other. *Synechococcaceae* in Module-1 and Module-3 affiliated with *Cyanobacteria* is one of the most important members of prokaryotic autotrophic picoplankton^58^,^84^. ACK-M1 (acI-A) and C111 (members of acIV) are heterotrophic bacteria^85^. In early spring when there was no cyanobacterial bloom, the growth of dominant ACK-M1 (acI-A) and C111 (acIV) might depend on metabolites from *Synechococcaceae.* Thus, they might be highly correlated in abundance, which agreed with the highly connected status between Module-1 and Module-3 in the network. But cyanobacterial bloom species such as *Microcystis* spp. gradually developed into dominant species in summer and led to massive death of other microbes. This possibly broke the original co-occurrence between ACK-M1, C111 and *Synechococcaceae.*, which can explain the later separation of Module-1 and Module-3. In winter, temperature fell and cyanobacterial bloom fade away. The aquatic ecosystem restored to the “original status”, which was demonstrated as the recombination of Module-1 and Module-3. The variation of relationship between Module-1 and Module-3 verifies the reliability of our co-occurrence network to some extent. We may speculate that *Holophagaceae* and *Sinobacteraceae* in Module-1 and -3 may have similar trophic preference as actinobacterial taxa in aquatic systems as well.

In summary, this large-scale ultra-deep 16S rRNA sequencing study provided a comprehensive profile about the archaeal and bacterial community in Lake Taihu. The observed temporal variation demonstrated seasonal patterns and an annual periodicity. The synchrony of bacterial taxa and the change of co-occurrence networks between different species are helpful to reveal the influence of the cyanobacterial bloom on the microbial community in Lake Taihu. Based on this study, further works can be done in the future to gain better understanding of microbial ecosystem of the Lake. (1) Functional profile of microbial community by metagenome sequencing is necessary to unveil potential biological functions of archaeal or bacterial communities, especially in the restoration stage after bloom and the “original status” we discussed above. (2) Although we have explored the variations of the microbial community, driven factors contributing to the aforementioned variation patterns were not studied. Therefore, environmental factors should be involved in the future work in order to reveal the association between community variations and environment changes, such as changes in temperature, pH, dissolved oxygen and concentration of nitrogen and phosphorus. Especially, a quantitative measurement for the severity of water bloom is needed in order to associate it with community variation. (3) Due to preliminary filtering of dominant cyanobacterial species, an important part of microbial community, especially members of *Microcystis* that dominate in Lake Taihu during the summer and autumn, were missed. Perhaps, microbial community assemblage of carpet-like mucilaginous cyanobacterial aggregates^86^ in Lake Taihu is a good target for future experiments, since it is a kind of aggregation of *Cyanobacteria* and other taxa that are highly associated with each other and present real symbiosis relationships.

## Additional Information

**How to cite this article**: Li, J. *et al.* Annual periodicity in planktonic bacterial and archaeal community composition of eutrophic Lake Taihu. *Sci. Rep.* **5**, 15488; doi: 10.1038/srep15488 (2015).

## Supplementary Material

Supplementary Information

Supplementary Information

## Figures and Tables

**Table 1:** Sampling sites and months in Lake Taihu.

Sampling Site	Coordinate	Sampling Month	Date
ML	120°10'23″ E, 31°28'8″ N	Jan.	2012.01.11–2012.01.12
SZ	120°14'46″ E, 31°22 ‘44″ N	Mar.	2012.02.07–2012.03.08
SD	120°21'58″ E, 31°25'45″ N	Apr.	2012.04.09–2012.04.10
MS	120°15'33″ E, 31°15'8″ N	May	2012.05.08–2012.05.09
XH	120°23'56″ E, 31°10'40″ N	Jun.	2012.06.05–2012.06.07
XS	120°10'55″ E, 31°8'41″ N	Aug.	2012.08.11–2012.08.12
XM	120°7'19″ E, 30°58'35″ N	Sep.	2012.09.13–2012.09.14
DL	120°0’ 42″E, 31°8'11″ N	Oct.	2012.10.18–2012.10.19
DP	119°57'14″ E, 31°18'11″ N	Dec.	2012.12.12–2012.12.13
