# Supplementary material for: Annual periodicity in planktonic bacterial and archaeal community composition of eutrophic Lake Taihu
Source: Sci Rep. 2015 Oct 27;5:15488. doi: 10.1038/srep15488 (PMC4621408; doi:10.1038/srep15488)

# Annual periodicity in planktonic bacterial and archaeal community composition of eutrophic Lake Taihu

Junfeng Li [1#], Junyi Zhang[2,3#], Liyang Liu [1], Yucai Fan[2], Lianshuo Li[1], Yunfeng Yang[4], Zuhong Lu[2,5,*], Xuegong Zhang[1*]

[1] MOE Key Lab of Bioinformatics; Bioinformatics Division/Center for Synthetic and Systems Biology, TNLIST and Department of Automation, Tsinghua University, Beijing, China.

[2] State Key Lab for Bioelectronics, School of Biological Science and Medical Engineering, Southeast University, Nanjing, China.

[3] Wuxi Environmental Monitoring Centre, Wuxi, China

[4] State Key Joint Laboratory of Environment Simulation and Pollution Control, School of Environment, Tsinghua University, Beijing, China

[5] Department of Biomedical Engineering, Peking University, Beijing, China

[#] These authors contributed equally to this work.

[*] To whom correspondence should be addressed.

Emails: XZ: zhangxg@tsinghua.edu.cn, Tel: (8610)62794919; ZL: zhlu@seu.edu.cn, Tel. (8625)83793779

**Figure S1. α-diversity of archaeal community and bacterial community.** α-diversity was evaluated by both "observed species" and "PD_whole_tree" metrics defined in QIIME. Each curve corresponds to a sample (totally 81 samples). **(A)** "observed species" within archaeal community; **(B)** "PD_whole_tree" metric of archaeal community; **(C)** "observed species" within bacterial community; **(D)** "PD_whole_tree" metric of bacterial community.

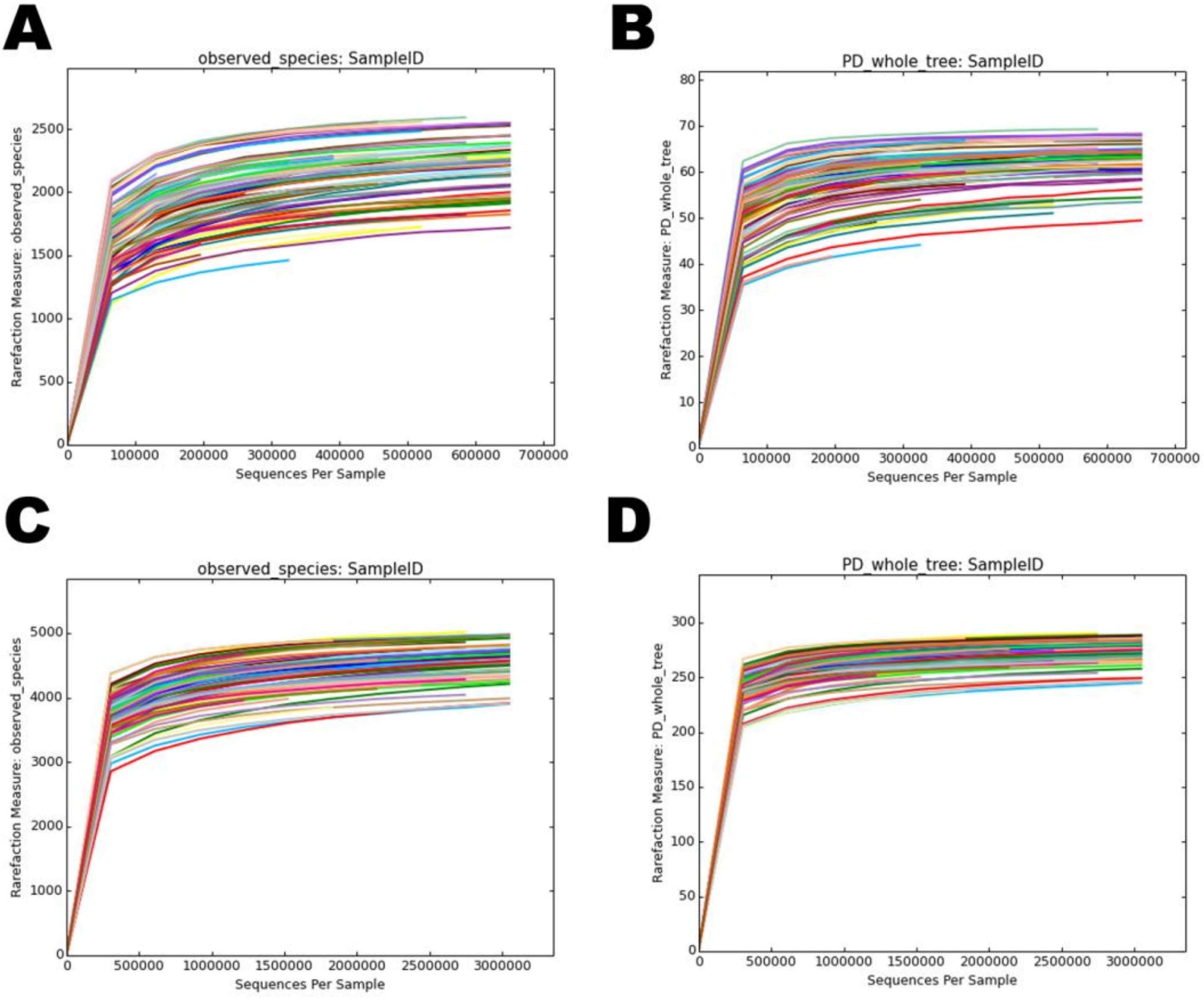

**Figure S2. Differential bacterial taxa between stages grouped according to UPGMA result.** Columns correspond to samples; rows correspond to significantly different taxa between two stages. Annotations of taxa were generated from Greengenes database. Prefix of annotations denotes taxonomy level, i.e. “k__” stands for “kingdom” and “g__” stands for “genus”. **(A)** Mar.-Apr. stage VS May.-Jun. stage; **(B)** May-Jun. stage VS Aug.-Oct. stage; **(C)** Aug.-Oct. stage VS Dec.-Jan. stage; **(D)** Dec.-Jan. stage VS Mar.-Apr. stage.

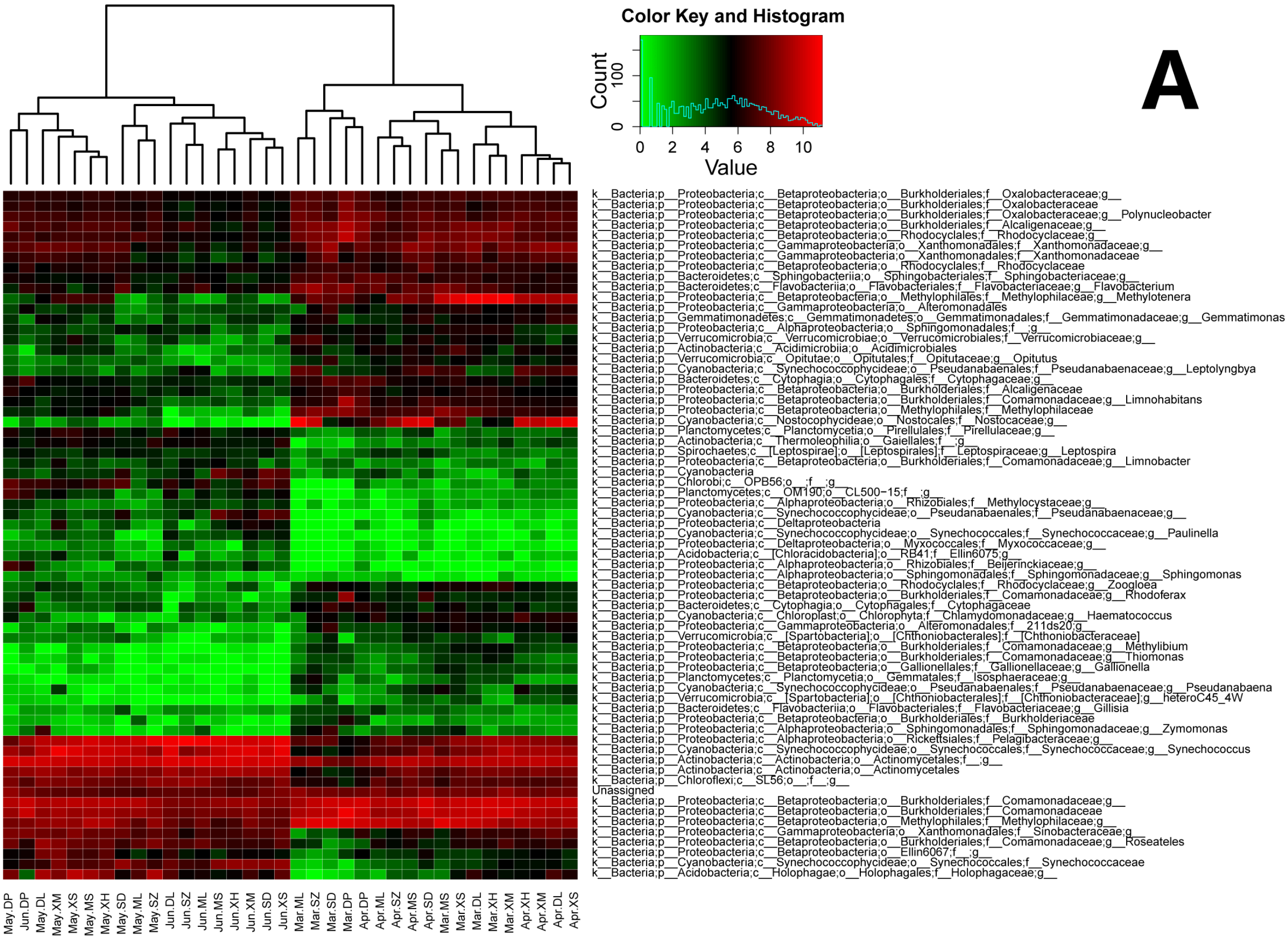

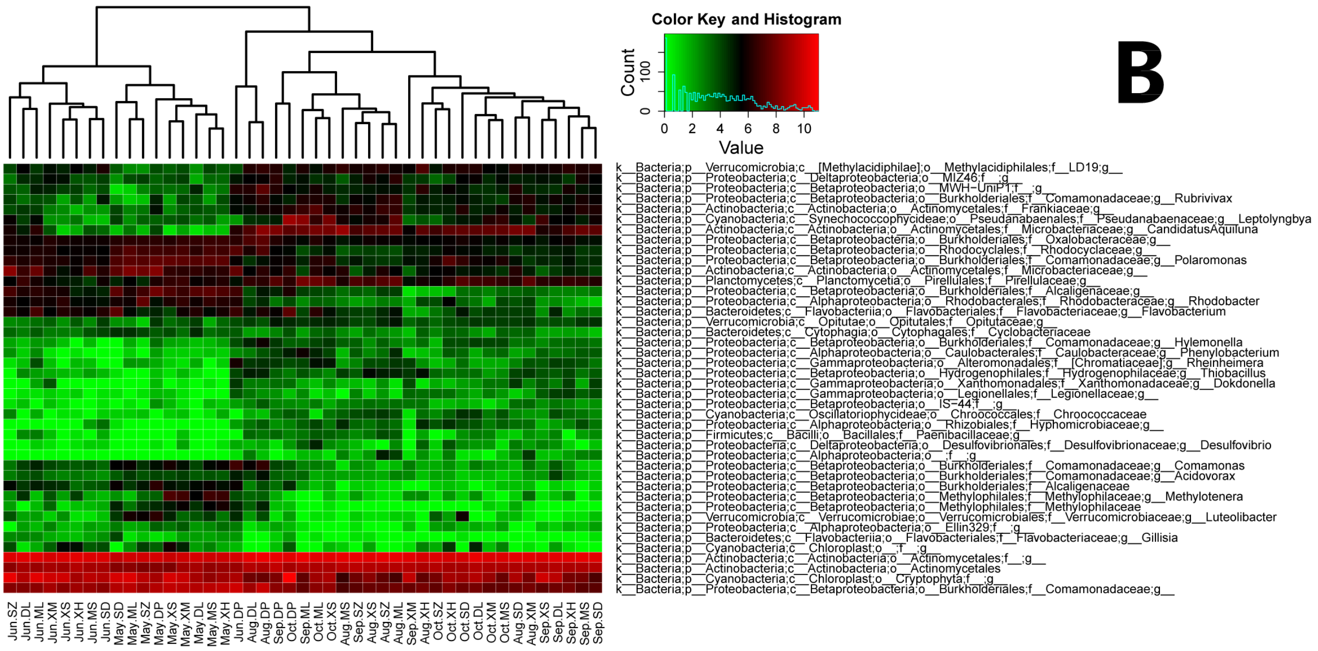
B
Color Key and Histogram
Count
0
100
0
2
4
6
8
10
Value
k__Bacteria;p__Verrucomicrobia;c__[Methylacidiphilae];o__Methylacidiphilales;f__LD19;g__
k__Bacteria;p__Proteobacteria;c__Deltaproteobacteria;o__MIZ46;f__;g__
k__Bacteria;p__Proteobacteria;c__Betaproteobacteria;o__MWH−UniP1;f__;g__
k__Bacteria;p__Proteobacteria;c__Betaproteobacteria;o__Burkholderiales;f__Comamonadaceae;g__Rubrivivax
k__Bacteria;p__Actinobacteria;c__Actinobacteria;o__Actinomycetales;f__Frankiaceae;g__
k__Bacteria;p__Cyanobacteria;c__Synechococcophycideae;o__Pseudanabaenales;f__Pseudanabaenaceae;g__Leptolyngbya
k__Bacteria;p__Actinobacteria;c__Actinobacteria;o__Actinomycetales;f__Microbacteriaceae;g__CandidatusAquiluna
k__Bacteria;p__Proteobacteria;c__Betaproteobacteria;o__Burkholderiales;f__Oxalobacteraceae;g__
k__Bacteria;p__Proteobacteria;c__Betaproteobacteria;o__Rhodocyclales;f__Rhodocyclaceae;g__
k__Bacteria;p__Proteobacteria;c__Betaproteobacteria;o__Burkholderiales;f__Comamonadaceae;g__Polaromonas
k__Bacteria;p__Actinobacteria;c__Actinobacteria;o__Actinomycetales;f__Microbacteriaceae;g__
k__Bacteria;p__Planctomycetes;c__Planctomycetia;o__Pirellulales;f__Pirellulaceae;g__
k__Bacteria;p__Proteobacteria;c__Betaproteobacteria;o__Burkholderiales;f__Alcaligenaceae;g__
k__Bacteria;p__Proteobacteria;c__Alphaproteobacteria;o__Rhodobacterales;f__Rhodobacteraceae;g__Rhodobacter
k__Bacteria;p__Bacteroidetes;c__Flavobacteriia;o__Flavobacteriales;f__Flavobacteriaceae;g__Flavobacterium
k__Bacteria;p__Verrucomicrobia;c__Opitutae;o__Opitutales;f__Opitutaceae;g__
k__Bacteria;p__Bacteroidetes;c__Cytophagia;o__Cytophagales;f__Cyclobacteriaceae
k__Bacteria;p__Proteobacteria;c__Betaproteobacteria;o__Burkholderiales;f__Comamonadaceae;g__Hylemonella
k__Bacteria;p__Proteobacteria;c__Alphaproteobacteria;o__Caulobacterales;f__Caulobacteraceae;g__Phenylobacterium
k__Bacteria;p__Proteobacteria;c__Gammaproteobacteria;o__Alteromonadales;f__[Chromatiaceae];g__Rheinheimera
k__Bacteria;p__Proteobacteria;c__Betaproteobacteria;o__Hydrogenophilales;f__Hydrogenophilaceae;g__Thiobacillus
k__Bacteria;p__Proteobacteria;c__Gammaproteobacteria;o__Xanthomonadales;f__Xanthomonadaceae;g__Dokdonella
k__Bacteria;p__Proteobacteria;c__Gammaproteobacteria;o__Legionellales;f__Legionellaceae;g__
k__Bacteria;p__Proteobacteria;c__Betaproteobacteria;o__IS−44;f__;g__
k__Bacteria;p__Cyanobacteria;c__Oscillatoriophycideae;o__Chroococcales;f__Chroococcaceae
k__Bacteria;p__Proteobacteria;c__Alphaproteobacteria;o__Rhizobiales;f__Hyphomicrobiaceae;g__
k__Bacteria;p__Firmicutes;c__Bacilli;o__Bacillales;f__Paenibacillaceae;g__
k__Bacteria;p__Proteobacteria;c__Deltaproteobacteria;o__Desulfovibrionales;f__Desulfovibrionaceae;g__Desulfovibrio
k__Bacteria;p__Proteobacteria;c__Alphaproteobacteria;o__;f__;g__
k__Bacteria;p__Proteobacteria;c__Betaproteobacteria;o__Burkholderiales;f__Comamonadaceae;g__Comamonas
k__Bacteria;p__Proteobacteria;c__Betaproteobacteria;o__Burkholderiales;f__Comamonadaceae;g__Acidovorax
k__Bacteria;p__Proteobacteria;c__Betaproteobacteria;o__Burkholderiales;f__Alcaligenaceae
k__Bacteria;p__Proteobacteria;c__Betaproteobacteria;o__Methylophilales;f__Methylophilaceae;g__Methylotenera
k__Bacteria;p__Proteobacteria;c__Betaproteobacteria;o__Methylophilales;f__Methylophilaceae
k__Bacteria;p__Verrucomicrobia;c__Verrucomicrobiae;o__Verrucomicrobiales;f__Verrucomicrobiaceae;g__Luteolibacter
k__Bacteria;p__Proteobacteria;c__Alphaproteobacteria;o__Ellin329;f__;g__
k__Bacteria;p__Bacteroidetes;c__Flavobacteriia;o__Flavobacteriales;f__Flavobacteriaceae;g__Gillisia
k__Bacteria;p__Cyanobacteria;c__Chloroplast;o__;f__;g__
k__Bacteria;p__Actinobacteria;c__Actinobacteria;o__Actinomycetales;f__;g__
k__Bacteria;p__Actinobacteria;c__Actinobacteria;o__Actinomycetales
k__Bacteria;p__Cyanobacteria;c__Chloroplast;o__Cryptophyta;f__;g__
k__Bacteria;p__Proteobacteria;c__Betaproteobacteria;o__Burkholderiales;f__Comamonadaceae;g__
Jun.SZ
Jun.DL
Jun.ML
Jun.XM
Jun.XS
Jun.XH
Jun.MS
Jun.SD
May.SD
May.ML
May.SZ
May.DP
May.XS
May.XM
May.DL
May.MS
May.XH
Jun.DP
Aug.DL
Aug.DP
Sep.DP
Oct.DP
Sep.ML
Oct.ML
Oct.XS
Aug.MS
Sep.SZ
Aug.XS
Aug.SZ
Aug.ML
Sep.XM
Aug.XH
Oct.SZ
Oct.XH
Oct.SD
Oct.DL
Oct.XM
Oct.MS
Aug.SD
Aug.XM
Sep.XS
Sep.DL
Sep.XH
Sep.MS
Sep.SD

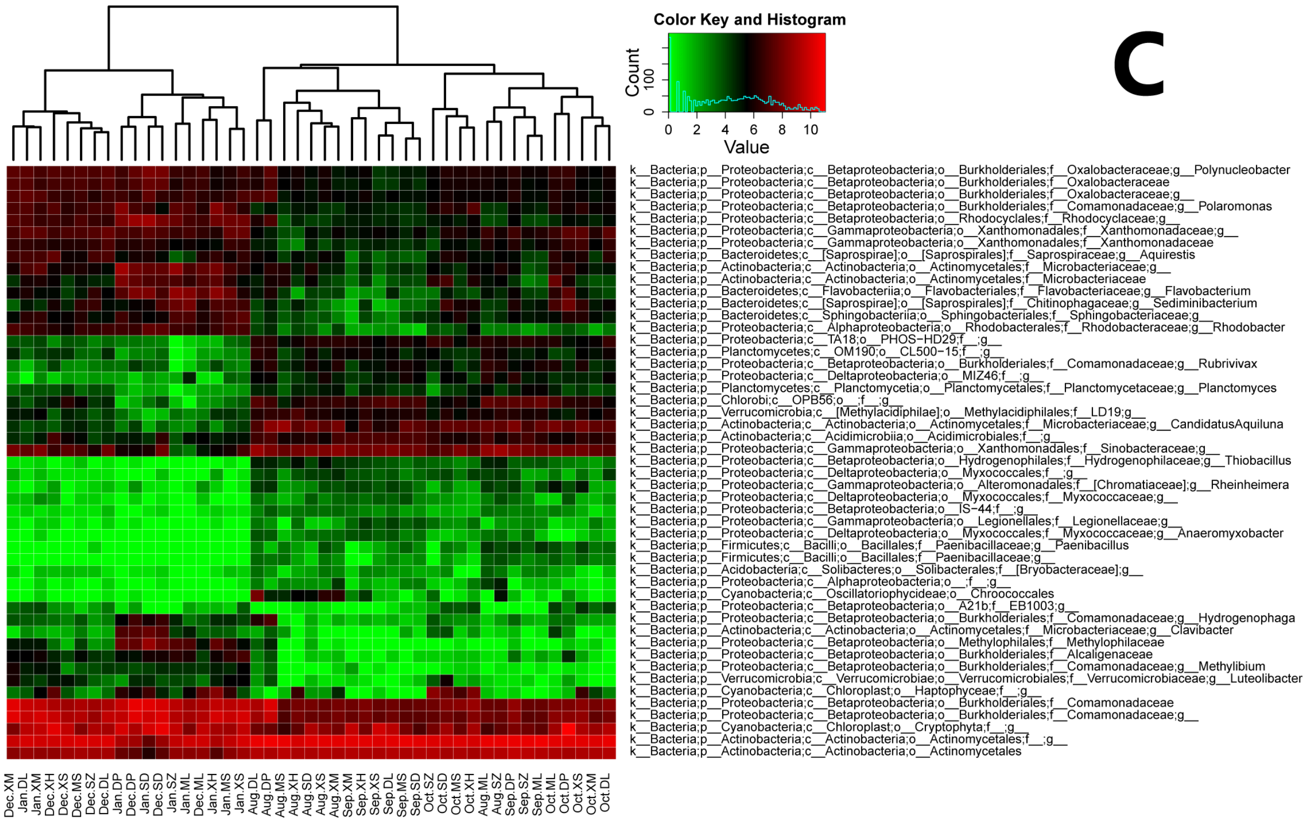
C
Color Key and Histogram
Count
0
100
0
2
4
6
8
10
Value
k__Bacteria;p__Proteobacteria;c__Betaproteobacteria;o__Burkholderiales;f__Oxalobacteraceae;g__Polynucleobacter
k__Bacteria;p__Proteobacteria;c__Betaproteobacteria;o__Burkholderiales;f__Oxalobacteraceae
k__Bacteria;p__Proteobacteria;c__Betaproteobacteria;o__Burkholderiales;f__Oxalobacteraceae;g__
k__Bacteria;p__Proteobacteria;c__Betaproteobacteria;o__Burkholderiales;f__Comamonadaceae;g__Polaromonas
k__Bacteria;p__Proteobacteria;c__Betaproteobacteria;o__Rhodocyclales;f__Rhodocyclaceae;g__
k__Bacteria;p__Proteobacteria;c__Gammaproteobacteria;o__Xanthomonadales;f__Xanthomonadaceae;g__
k__Bacteria;p__Proteobacteria;c__Gammaproteobacteria;o__Xanthomonadales;f__Xanthomonadaceae
k__Bacteria;p__Bacteroidetes;c__[Saprospirae];o__[Saprospirales];f__Saprospiraceae;g__Aquirestis
k__Bacteria;p__Actinobacteria;c__Actinobacteria;o__Actinomycetales;f__Microbacteriaceae;g__
k__Bacteria;p__Actinobacteria;c__Actinobacteria;o__Actinomycetales;f__Microbacteriaceae
k__Bacteria;p__Bacteroidetes;c__Flavobacteriia;o__Flavobacteriales;f__Flavobacteriaceae;g__Flavobacterium
k__Bacteria;p__Bacteroidetes;c__[Saprospirae];o__[Saprospirales];f__Chitinophagaceae;g__Sediminibacterium
k__Bacteria;p__Bacteroidetes;c__Sphingobacteriia;o__Sphingobacteriales;f__Sphingobacteriaceae;g__
k__Bacteria;p__Proteobacteria;c__Alphaproteobacteria;o__Rhodobacterales;f__Rhodobacteraceae;g__Rhodobacter
k__Bacteria;p__Proteobacteria;c__TA18;o__PHOS−HD29;f__;g__
k__Bacteria;p__Planctomycetes;c__OM190;o__CL500−15;f__;g__
k__Bacteria;p__Proteobacteria;c__Betaproteobacteria;o__Burkholderiales;f__Comamonadaceae;g__Rubrivivax
k__Bacteria;p__Proteobacteria;c__Deltaproteobacteria;o__MIZ46;f__;g__
k__Bacteria;p__Planctomycetes;c__Planctomycetia;o__Planctomycetales;f__Planctomycetaceae;g__Planctomyces
k__Bacteria;p__Chlorobi;c__OPB56;o__;f__;g__
k__Bacteria;p__Verrucomicrobia;c__[Methylacidiphilae];o__Methylacidiphilales;f__LD19;g__
k__Bacteria;p__Actinobacteria;c__Actinobacteria;o__Actinomycetales;f__Microbacteriaceae;g__CandidatusAquiluna
k__Bacteria;p__Actinobacteria;c__Acidimicrobiia;o__Acidimicrobiales;f__;g__
k__Bacteria;p__Proteobacteria;c__Gammaproteobacteria;o__Xanthomonadales;f__Sinobacteraceae;g__
k__Bacteria;p__Proteobacteria;c__Betaproteobacteria;o__Hydrogenophilales;f__Hydrogenophilaceae;g__Thiobacillus
k__Bacteria;p__Proteobacteria;c__Deltaproteobacteria;o__Myxococcales;f__;g__
k__Bacteria;p__Proteobacteria;c__Gammaproteobacteria;o__Alteromonadales;f__[Chromatiaceae];g__Rheinheimera
k__Bacteria;p__Proteobacteria;c__Deltaproteobacteria;o__Myxococcales;f__Myxococcaceae;g__
k__Bacteria;p__Proteobacteria;c__Betaproteobacteria;o__IS−44;f__;g__
k__Bacteria;p__Proteobacteria;c__Gammaproteobacteria;o__Legionellales;f__Legionellaceae;g__
k__Bacteria;p__Proteobacteria;c__Deltaproteobacteria;o__Myxococcales;f__Myxococcaceae;g__Anaeromyxobacter
k__Bacteria;p__Firmicutes;c__Bacilli;o__Bacillales;f__Paenibacillaceae;g__Paenibacillus
k__Bacteria;p__Firmicutes;c__Bacilli;o__Bacillales;f__Paenibacillaceae;g__
k__Bacteria;p__Acidobacteria;c__Solibacteres;o__Solibacterales;f__[Bryobacteraceae];g__
k__Bacteria;p__Proteobacteria;c__Alphaproteobacteria;o__;f__;g__
k__Bacteria;p__Cyanobacteria;c__Oscillatoriophycideae;o__Chroococcales
k__Bacteria;p__Proteobacteria;c__Betaproteobacteria;o__A21b;f__EB1003;g__
k__Bacteria;p__Proteobacteria;c__Betaproteobacteria;o__Burkholderiales;f__Comamonadaceae;g__Hydrogenophaga
k__Bacteria;p__Actinobacteria;c__Actinobacteria;o__Actinomycetales;f__Microbacteriaceae;g__Clavibacter
k__Bacteria;p__Proteobacteria;c__Betaproteobacteria;o__Methylophilales;f__Methylophilaceae
k__Bacteria;p__Proteobacteria;c__Betaproteobacteria;o__Burkholderiales;f__Alcaligenaceae
k__Bacteria;p__Proteobacteria;c__Betaproteobacteria;o__Burkholderiales;f__Comamonadaceae;g__Methylibium
k__Bacteria;p__Verrucomicrobia;c__Verrucomicrobiae;o__Verrucomicrobiales;f__Verrucomicrobiaceae;g__Luteolibacter
k__Bacteria;p__Cyanobacteria;c__Chloroplast;o__Haptophyceae;f__;g__
k__Bacteria;p__Proteobacteria;c__Betaproteobacteria;o__Burkholderiales;f__Comamonadaceae
k__Bacteria;p__Proteobacteria;c__Betaproteobacteria;o__Burkholderiales;f__Comamonadaceae;g__
k__Bacteria;p__Cyanobacteria;c__Chloroplast;o__Cryptophyta;f__;g__
k__Bacteria;p__Actinobacteria;c__Actinobacteria;o__Actinomycetales;f__;g__
k__Bacteria;p__Actinobacteria;c__Actinobacteria;o__Actinomycetales
Dec.XM
Jan.DL
Jan.XM
Dec.XH
Dec.XS
Dec.MS
Dec.SZ
Dec.DL
Jan.DP
Dec.DP
Jan.SD
Dec.SD
Jan.SZ
Jan.ML
Dec.ML
Jan.XH
Jan.MS
Jan.XS
Aug.DL
Aug.DP
Aug.MS
Aug.XH
Aug.SD
Aug.XS
Aug.XM
Sep.XM
Sep.XH
Sep.XS
Sep.DL
Sep.MS
Sep.SD
Oct.SZ
Oct.SD
Oct.MS
Oct.XH
Aug.ML
Aug.SZ
Sep.DP
Sep.SZ
Sep.ML
Oct.ML
Oct.DP
Oct.XS
Oct.XM
Oct.DL

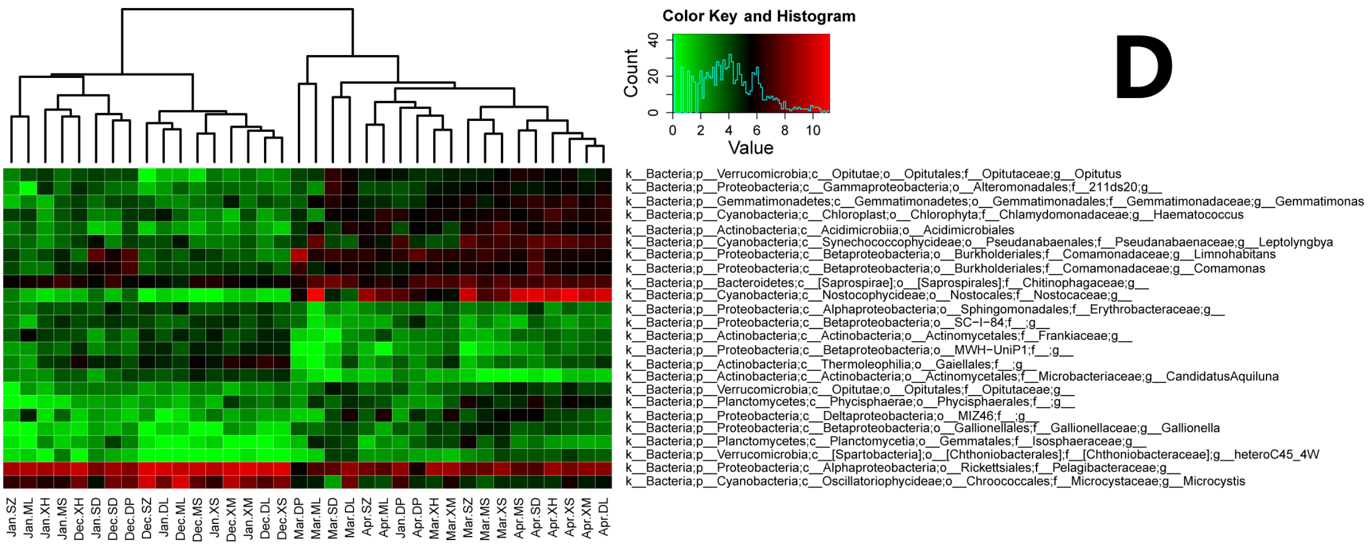
Color Key and Histogram
Count
0
20
40
0 2 4 6 8 10
Value
D
k__Bacteria;p__Verrucomicrobia;c__Opitutae;o__Opitutales;f__Opitutaceae;g__Opitutus
k__Bacteria;p__Proteobacteria;c__Gammaproteobacteria;o__Alteromonadales;f__211ds20;g__
k__Bacteria;p__Gemmatimonadetes;c__Gemmatimonadetes;o__Gemmatimonadales;f__Gemmatimonadaceae;g__Gemmatimonas
k__Bacteria;p__Cyanobacteria;c__Chloroplast;o__Chlorophyta;f__Chlamydomonadaceae;g__Haematococcus
k__Bacteria;p__Actinobacteria;c__Acidimicrobiia;o__Acidimicrobiales
k__Bacteria;p__Cyanobacteria;c__Synechococcophycideae;o__Pseudanabaenales;f__Pseudanabaenaceae;g__Leptolyngbya
k__Bacteria;p__Proteobacteria;c__Betaproteobacteria;o__Burkholderiales;f__Comamonadaceae;g__Limnohabitans
k__Bacteria;p__Proteobacteria;c__Betaproteobacteria;o__Burkholderiales;f__Comamonadaceae;g__Comamonas
k__Bacteria;p__Bacteroidetes;c__[Saprospirae];o__[Saprospirales];f__Chitinophagaceae;g__
k__Bacteria;p__Cyanobacteria;c__Nostocophycideae;o__Nostocales;f__Nostocaceae;g__
k__Bacteria;p__Proteobacteria;c__Alphaproteobacteria;o__Sphingomonadales;f__Erythrobacteraceae;g__
k__Bacteria;p__Proteobacteria;c__Betaproteobacteria;o__SC−I−84;f__;g__
k__Bacteria;p__Actinobacteria;c__Actinobacteria;o__Actinomycetales;f__Frankiaceae;g__
k__Bacteria;p__Proteobacteria;c__Betaproteobacteria;o__MWH−UniP1;f__;g__
k__Bacteria;p__Actinobacteria;c__Thermoleophilia;o__Gaiellales;f__;g__
k__Bacteria;p__Actinobacteria;c__Actinobacteria;o__Actinomycetales;f__Microbacteriaceae;g__CandidatusAquiluna
k__Bacteria;p__Verrucomicrobia;c__Opitutae;o__Opitutales;f__Opitutaceae;g__
k__Bacteria;p__Planctomycetes;c__Phycisphaerae;o__Phycisphaerales;f__;g__
k__Bacteria;p__Proteobacteria;c__Deltaproteobacteria;o__MIZ46;f__;g__
k__Bacteria;p__Proteobacteria;c__Betaproteobacteria;o__Gallionellales;f__Gallionellaceae;g__Gallionella
k__Bacteria;p__Planctomycetes;c__Planctomycetia;o__Gemmatales;f__Isosphaeraceae;g__
k__Bacteria;p__Verrucomicrobia;c__[Spartobacteria];o__[Chthoniobacterales];f__[Chthoniobacteraceae];g__heteroC45_4W
k__Bacteria;p__Proteobacteria;c__Alphaproteobacteria;o__Rickettsiales;f__Pelagibacteraceae;g__
k__Bacteria;p__Cyanobacteria;c__Oscillatoriophycideae;o__Chroococcales;f__Microcystaceae;g__Microcystis
Jan.SZ
Jan.ML
Jan.XH
Jan.MS
Dec.XH
Jan.SD
Dec.SD
Dec.DP
Dec.SZ
Jan.DL
Dec.ML
Dec.MS
Jan.XS
Dec.XM
Jan.XM
Dec.DL
Dec.XS
Mar.DP
Mar.ML
Mar.SD
Mar.DL
Apr.SZ
Apr.ML
Jan.DP
Apr.DP
Mar.XH
Mar.XM
Mar.SZ
Mar.MS
Mar.XS
Apr.MS
Apr.SD
Apr.XH
Apr.XS
Apr.XM
Apr.DL

**Figure S3. Temporal variation of Z-scores of K-means clustering centers.** Relative abundance of bacterial genera at different sample sites was transformed into Z-scores respectively with similar method described in Materials and Methods. Unsupervised clustering was conducted using K-means algorithm. Average Z-scores of the genera of each cluster are demonstrated in 9 figures, corresponding to 9 different sample sites. Intuitively, we can see two clusters whose relative abundances vary in opposite ways are quite consistent across different sites.

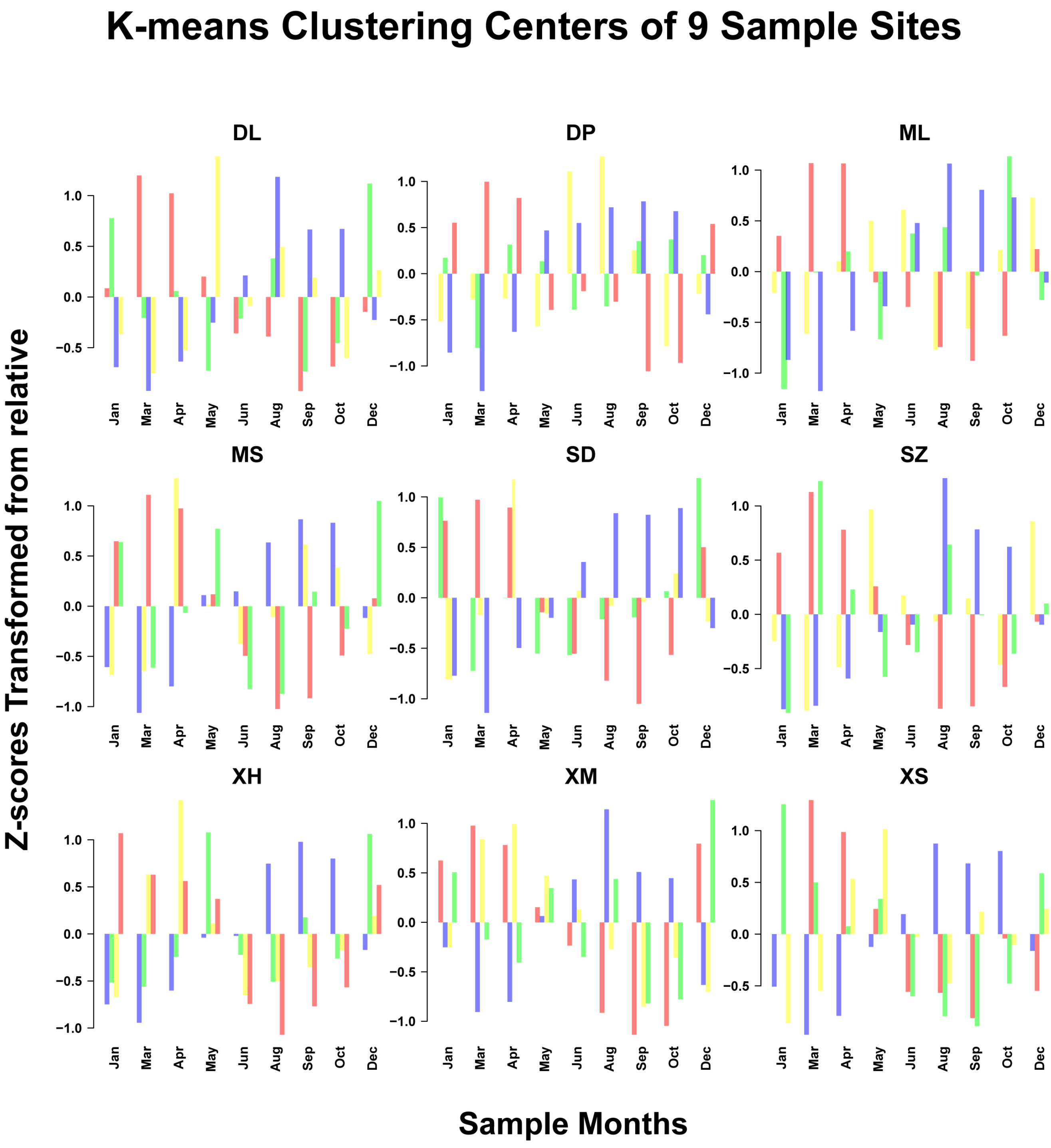

**Figure S4. Overview of the OTU co-occurrence networks of four stages.** We used MCODE to find network modules in Mar.-Apr. stage, as it had been reported to be the initiation period of harmful algal bloom in which temperature rose gradually. Top 10 modules ranked by ratio of edge number and node number were colored. OTUs within the modules were also colored in other three networks for tracing temporal variation of the modules.

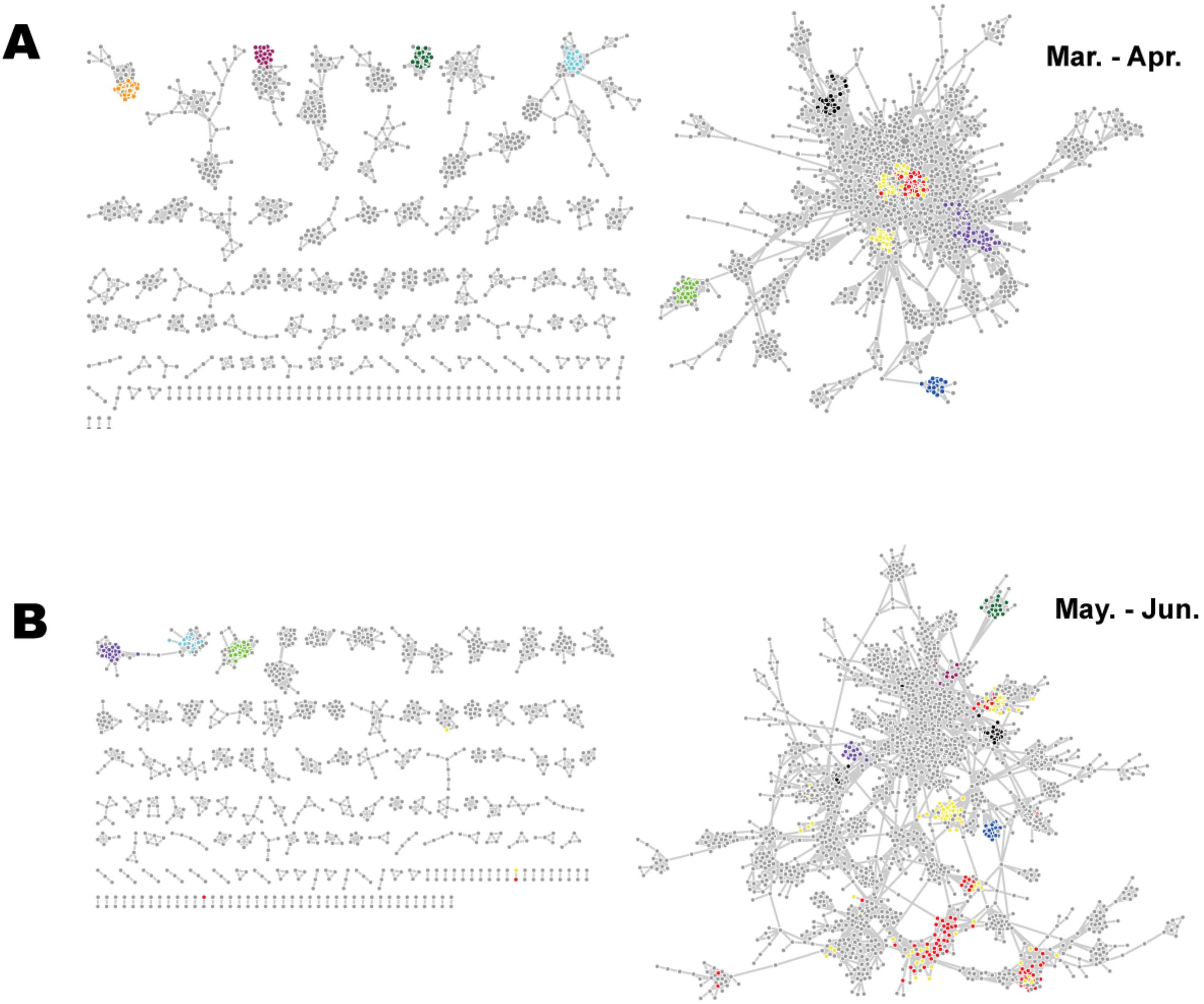

C

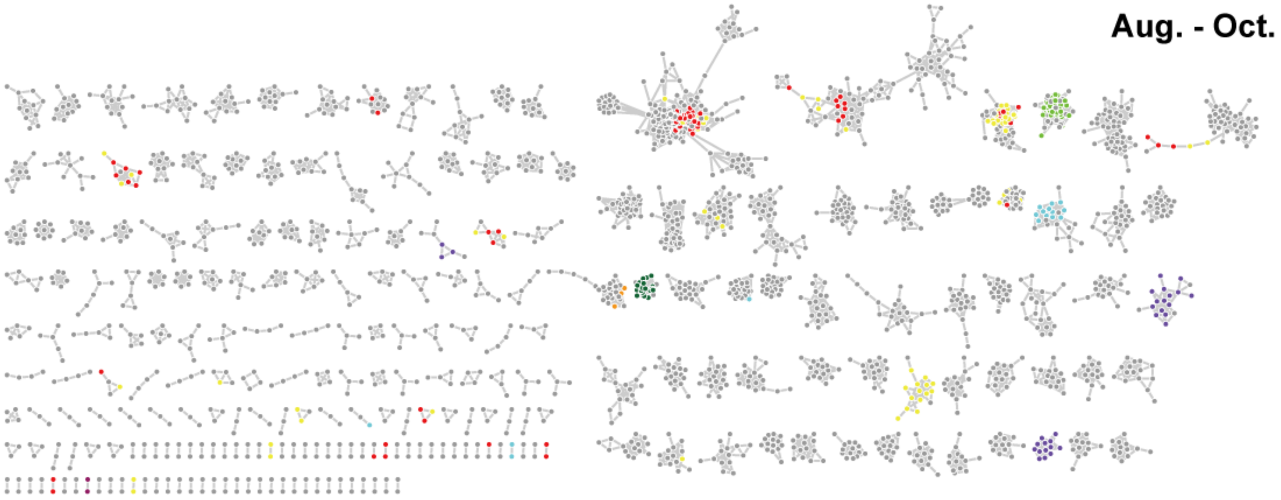


D

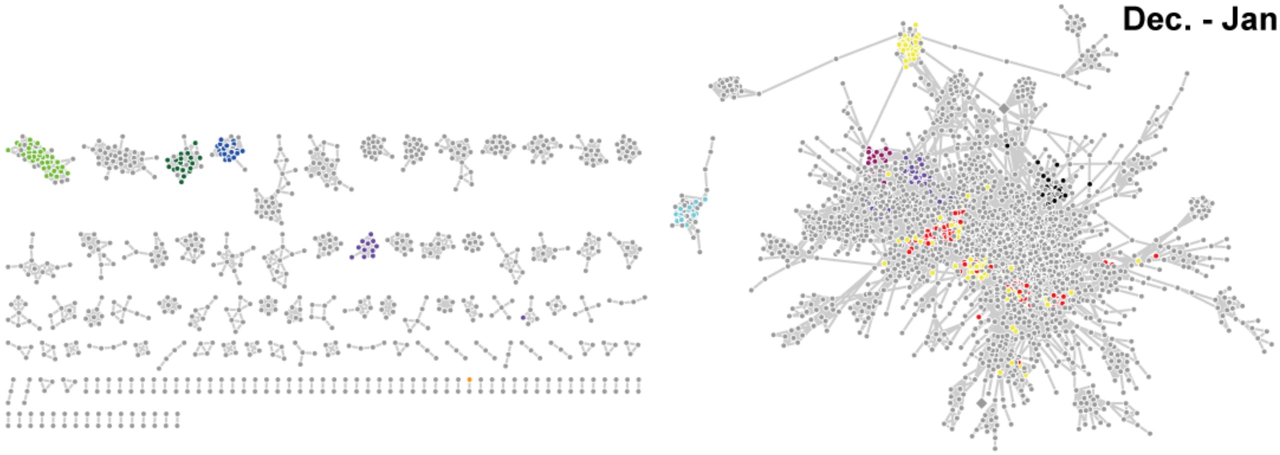

Supplement: Supplementary Information [file srep15488-s1.pdf]
